# The impact of HIV status and antiretroviral treatment on TB treatment outcomes of new tuberculosis patients attending co-located TB and ART services in South Africa: a retrospective cohort study

**DOI:** 10.1186/s12879-015-1275-3

**Published:** 2015-11-19

**Authors:** Mweete D. Nglazi, Linda-Gail Bekker, Robin Wood, Richard Kaplan

**Affiliations:** The Desmond Tutu HIV Centre, Institute of Infectious Disease and Molecular Medicine and the Department of Medicine, Faculty of Health Sciences, University of Cape Town, Cape Town, South Africa; International Union against Tuberculosis and Lung Disease, Paris, France; Burden of Disease Research Unit, South African Medical Research Council, Tygerberg Cape Town, South Africa

**Keywords:** Tuberculosis, HIV/AIDS, Treatment outcomes, Integrated TB/HIV care, TB treatment, Antiretroviral therapy

## Abstract

**Background:**

The implementation of collaborative TB-HIV services is challenging. We, therefore, assessed TB treatment outcomes in relation to HIV infection and antiretroviral therapy (ART) among TB patients attending a primary care service with co-located ART and TB clinics in Cape Town, South Africa.

**Methods:**

In this retrospective cohort study, all new TB patients aged ≥ 15 years who registered and initiated TB treatment between 1 October 2009 and 30 June 2011 were identified from an electronic database. The effects of HIV-infection and ART on TB treatment outcomes were analysed using a multinomial logistic regression model, in which treatment success was the reference outcome.

**Results:**

The 797 new TB patients included in the analysis were categorized as follows: HIV- negative, in 325 patients (40.8 %); HIV-positive on ART, in 339 patients (42.5 %) and HIV-positive not on ART, in 133 patients (16.7 %). Overall, bivariate analyses showed no significant difference in death and default rates between HIV-positive TB patients on ART and HIV-negative patients. Statistically significant higher mortality rates were found among HIV-positive patients not on ART compared to HIV-negative patients (unadjusted odds ratio (OR) 3.25; 95 % confidence interval (CI) 1.53–6.91). When multivariate analyses were conducted, the only significant difference between the patient categories on TB treatment outcomes was that HIV-positive TB patients not on ART had significantly higher mortality rates than HIV-negative patients (adjusted OR 4.12; 95 % CI 1.76–9.66). Among HIV-positive TB patients (*n* = 472), 28.2 % deemed eligible did not initiate ART in spite of the co-location of TB and ART services. When multivariate analyses were restricted to HIV-positive patients in the cohort, we found that being HIV-positive not on ART was associated with higher mortality (adjusted OR 7.12; 95 % CI 2.95–18.47) and higher default rates (adjusted OR 2.27; 95 % CI 1.15–4.47).

**Conclusions:**

There was no significant difference in death and default rates between HIV-positive TB patients on ART and HIV negative TB patients. Despite the co-location of services 28.2 % of 472 HIV-positive TB patients deemed eligible did not initiate ART. These patients had a significantly higher death and default rates.

## Background

Tuberculosis (TB) is a global epidemic, with an estimated 9.0 million new TB cases and 1.5 million deaths in 2013[1]. The global burden of TB falls heaviest on 22 low- and middle-income countries, including those in sub-Saharan Africa, where TB is fuelled by the HIV/AIDS epidemic [1, 2]. In South Africa, there is a high burden of both TB and HIV [1–3], and a high HIV-associated TB case fatality rate [4].

In high TB and HIV prevalence settings such as South Africa, patients accessing TB services can be broadly categorized as HIV-negative TB patients or HIV-positive TB patients. In terms of optimal case management, both patient groups require standardized anti-tuberculosis treatment, while HIV-positive TB patients also require trimethoprim-sulphamethoxazole (co-trimoxazole) prophylaxis and antiretroviral treatment (ART). ART results in a 64–95 % reduction in mortality risk [5] and should be initiated early after starting anti-tuberculosis treatment [6–8]. Compared to their HIV-negative peers, HIV-positive TB patients are a challenge to TB services, as they are more likely to have diagnostic delays, are more likely to be infectious for longer and, if not properly managed, may also have poorer TB treatment outcomes [9]. Therefore, HIV infection has the potential to profoundly impact on TB treatment outcomes and is often the main reason for failure to meet control targets in high HIV settings.

In order to control TB in high HIV prevalence settings, the World Health organization (WHO) has recommended TB/HIV collaborative activities, whose objectives are to create mechanisms for collaboration between TB and HIV/AIDS programs, reduce the burden of TB among people living with HIV and reduce the burden of HIV among TB patients [10]. The interpretation of this recommendation varies as demonstrated in a systematic review that described five different models of TB/HIV service integration in low-and middle- income countries, ranging from efficient referral between services, to collaboration between HIV and TB services in the same health facility, and to fully integrated services [11]. The ultimate goals of the models of integration are to reduce rates of mortality, default and relapse; improve rates of TB cure and to prevent rates of drug resistance. However, in practice challenges remain in the implementation of TB/HIV collaborative activities. For instance, ART uptake may be delayed due to patients having high CD4 counts, drug stockout, patients’ fear of drug interactions and overlapping side effects [12], fear of clinicians and national health policy [13]. In addition, the lack of integration of TB and HIV services at all levels in the health system may negatively impact the provision of care [14].

Numerous studies from sub-Saharan Africa have examined the impact of HIV infection on TB treatment outcomes. These studies have yielded mixed results. Some studies have demonstrated poorer TB treatment outcomes in HIV-positive TB patients when compared to HIV-negative TB patients [15–20], while others found that TB treatment outcomes did not differ between the two groups [21, 22]. In addition, the benefit of ART on TB treatment outcomes has been demonstrated in results from the above-cited randomized controlled trials [6–8] and observational studies [19, 23, 24]. Also, a recent systematic review of studies conducted in sub-Saharan Africa and elsewhere showed the benefit of ART on TB mortality, and indicated a 44 to 71 % reduction in TB mortality risk [25].

TB mortality among HIV-positive TB patients is one of the key indicators that measures the impact of collaborative TB/HIV activities [26]. With the advent of ART, it has become increasingly clear that HIV-positive TB patients are not a homogeneous group, and TB mortality during TB treatment will differ between HIV-positive TB patients on ART and those not on ART. However, to date, there has been no study on TB mortality in HIV-positive TB patients on ART relative to HIV-negative TB patients. Also, to date, there has been no study on TB mortality in HIV-positive TB patients not on ART relative to HIV-negative TB patients.

The aim of the study, therefore, was to establish whether there was a difference in the TB treatment outcomes (mortality and treatment default) in HIV-positive patients on ART compared to HIV-negative TB patients and also among HIV-positive patients not on ART compared to HIV-negative TB patients attending co-located TB services in Cape Town, South Africa. In addition, we aimed to examine impact of ART status and CD4 status on TB treatment outcomes among HIV-positive TB patients.

## Methods

### Population and design

This was a retrospective cohort study conducted at the Nyanga Community Health Centre. The Nyanga Community Health Centre is situated in the Klipfontein health sub-district, Cape Town, South Africa. An estimated 420, 000 people [27] live in this predominantly low-income urban community, which had an antenatal HIV-1 prevalence rate of 24 % in 2009 [28]. This nurse-run, doctor supported service provides TB and ART care in two separate clinics run by different health authorities but in the same building and has done so since the introduction of an ART service in 2008. The Cape Town City Health department was responsible for the TB services in this clinic while the Western Cape Provincial Department of Health introduced and managed the ART service.

While ART and TB services were not fully integrated in this clinic, the adherence support and the monitoring and evaluation systems were partially integrated and all patients starting ART or TB treatment were reviewed at a weekly multidisciplinary team meeting which was attended by TB and HIV staff. TB treatment was dispensed by trained TB nurses under the supervision of a clinician while ART was provided by a team comprised of two clinical nurse practitioners and a doctor. This study was conducted after the introduction of the ART services to the clinic.

### Inclusion and exclusion criteria

We included all new adult TB patients (aged ≥ 15 years) who registered and initiated TB treatment between 1 October 2009 and 30 June 2011. We excluded those for whom treatment outcomes were inconclusive (14 unknown and 14 transferred out) or who failed treatment (*n* = 20). We also excluded those for whom HIV status was unknown (*n* = 13).

### Data collection

Data from the Cape Town City Health electronic TB register (ETR.net) were compared with that from the Western Cape Department of Health’s electronic eKapa TB and ART database and any differences were resolved by reviewing patient folders and paper-based TB registers. The data are not freely available but permission for access was obtained from Cape Town City Health and Western Cape Department of Health. Variables for all patients meeting the inclusion criteria were obtained from the synchronized electronic TB register database. Variables included TB registration number, registration date, age, gender, HIV status, CD4 count at TB diagnosis, ART status and TB treatment outcome. Ethical approval for anonymized data collection was obtained from the Research Ethics Committees of the University of Cape Town and the International Union against Tuberculosis and Lung Disease.

### Outcome measures and definitions

For the purpose of this study, internationally recommended definitions for TB treatment outcomes (classified as either cured, treatment completed, died, defaulted or transferred out) [29] were used, in addition to the category “unknown”. Cure was defined as the presence of a negative sputum smear at the last month of treatment and at least on one other occasion during the course of treatment. Treatment completion referred to a patient who completed treatment, but for whom smear examination results were not complete enough to classify the patient as cured; or based on clinical, radiological and complementary examination criteria in those patients who did not produce sputum for a smear examination. Treatment success referred to the combined number of patients belonging to the categories “cured” and ‘treatment competed”. Death was defined as all-cause mortality occurring after TB diagnosis and before the end of treatment. A patient was defined as a defaulter when he or she did not collect medicines for two or more consecutive months.

### Anti-tuberculosis and antiretroviral therapy

Standardized anti-tuberculosis regimens for new adult TB cases consisted of a combination of rifampicin, isoniazid pyrazinamide and ethambutol during the two initial months (“intensive phase”) followed by rifampicin plus isoniazid during four months (“continuation phase”) [30].

Antiretroviral therapy was offered according to the South African ART guidelines that were updated once during the study period. Before April 2010, staff at both TB and ART services followed the 2004 South African ART guidelines which recommended that patients with CD4 counts of 50–200 cells/μL should delay ART until after 2 months of starting TB treatment, while those with a CD4 count <50 cells/μL or with serious co-morbidities should commence ART as soon as possible after at least 2 weeks of TB treatment [31]. After April 2010, the clinic staff were advised to adopt the 2010 South African ART guidelines, which recommended that TB patients with CD4 cell counts <100 cells/μL or WHO stage IV should commence ART within 2 weeks of eligibility, and those with CD4 cell counts ≤350 cells/μl should commence ART within 2–8 weeks of starting TB treatment [32].

### Statistical analysis

Patient characteristics were tabulated and stratified by HIV and ART status (categorized as HIV-positive on ART, HIV-positive not on ART and HIV-negative). The chi-square test or Fisher’s exact test were used to compare the distribution of categorical variables. The Kruskal-Wallis test or Wilcoxon rank-sum was used to compare the distribution of continuous variables.

The effects of HIV status, ART status and CD4 count on TB treatment outcome were examined using multinomial logistic regression models. In the multinomial logistic regression analysis, “treatment success” was the reference category for the dependent variable, being compared with the other categories (default versus treatment success; death versus treatment success). Independent variables were as follows: age (continuous); gender (male or female); type of TB (smear-negative pulmonary tuberculosis, smear-positive pulmonary tuberculosis or extra-pulmonary tuberculosis); CD4 count (<50, 50–199, 200–349 or ≥ 350 cells/ μL); HIV status (HIV-positive on ART, HIV-positive not on ART or HIV-negative) and ART status (HIV-positive on ART or HIV-positive not on ART).

All analyses were performed using STATA 12.1 (StataCorp, College Station, Texas, USA), and the level of significance was set at *P* <0.05. 95 % confidence intervals were also calculated throughout.

## Results

**Fig. 1:**
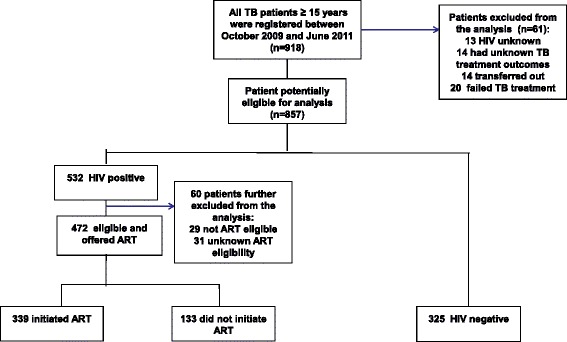
Flow diagram showing the HIV status and uptake of antiretroviral treatment for new TB patients ≥ 15 years who were registered between October 2009 and June 2011

A total of 918 new TB patients ≥ 15 years were registered between October 2009 and June 2011 (Fig. 1). The new TB patients (*n* = 61) for whom HIV status was unknown; tuberculosis treatment completion status was inconclusive (unknown, transferred out) or who failed tuberculosis treatment were exclude from the analysis (Fig. 1). We also excluded 60 patient categorized as follows: not eligible for ART, in 29 and unknown ART eligibility in 31 patients. This left 797 patients who formed the patient cohort in the study. HIV status in this sample was categorized as follows: HIV- negative, in 325 patients (40.8 %); HIV-positive on ART, in 339 patients (42.5 %) and HIV-positive not on ART, in 133 patients (16.7 %).

The proportion of patients who were female was higher among patients categorized as HIV-positive on ART and HIV positive not on ART (60.8 % and 60.2 %, respectively) (Table 1). The proportion of patients who had smear-positive TB was higher among those HIV-negative (*P* < 0.001). In contrast, no significant differences were found in the distribution of age for each category. The death rate was higher among HIV-positive not on ART compared to the other categories (*P* < 0.001) (Table 1).

**Table 1:** Patient characteristics by HIV status among 797 new TB patients ≥ 15 years who were registered between October 2009 and June 2011

	Total N (%)	HIV positive on ART N (%)	HIV positive not on ART N (%)	HIV negative N (%)	Statistical test	*P*-value
Age						
Median, IQR	33 (26–42)	33 (28–39)	33 (27–37)	32 (23–48)	Kruskal-Wallis	0.332
Gender						
Female	408 (51.2)	206 (60.8)	80 (60.2)	122 (37.5)	Chi-square	<0.001
Male	389 (48.8)	133 (39.2)	53 (39.9)	203 (62.5)		
Type of TB						
Smear-negative PTB	242 (30.4)	109 (32.2)	52 (39.1)	81 (24.9)	Chi-square	<0.001
Smear-positive PTB	357 (44.8)	104 (30.7)	48 (36.1)	205 (63.1)		
EPTB	198 (24.8)	126 (37.2)	33 (24.8)	39 (12.0)		
CD4 count, cells/μL						
Median, IQR	144 (74–249)	123 (66–203)	223.5 (109–386)	-	Wilcoxon rank-sum	<0.0001
<200	302 (65.0)	247 (73.7)	55 (42.3)	-	Chi-square	<0.001
200–349	115 (24.7)	77 (23.0)	38 (29.2)	-		
350-499	30 (6.5)	7 (2.1)	23 (17.7)	-		
≥500	18 (3.9)	4 (1.2)	14 (10.8)	-		
TB treatment outcome						
Success^a^	668 (83.8)	299 (88.2)	96 (72.2)	273 (84.0)	Chi-square	<0.001
Defaulted	88 (11.0)	29 (8.6)	21 (15.8)	38 (11.7)		
Died	41 (5.1)	11 (3.2)	16 | (12.0)	14 (4.3)		
Total	797 (100.0)	339 (42.5)	133 (16.7)	325 (40.8)		-

*IQR* interquartile range

Percentages may not add up to 100 due to rounding

^a^Defined as cured and treatment completed

Among HIV-positive TB patients (*n* = 472), the median CD4 count was higher in patients categorized HIV-positive not on ART than HIV-positive on ART (*P* < 0.0001) (Table 1). Just over one quarter (28.2 %) of HIV-positive TB patients deemed eligible did not initiate ART during their TB treatment (Fig. 1).

The findings of multinomial logistic regression analysis of the factors associated with treatment outcomes in new TB patients can be found in Table 2. The odds ratio (OR) for death in relation to treatment success was found to be significantly higher in patients HIV positive not on ART (adjusted OR 4.12; 95 % confidence interval (CI) 1.76–9.66) than in those HIV-negative. Increased age was associated with higher death rates in relation to treatment success (adjusted OR 1.04; 95 % CI 1.02–1.07) but reduced default rates in relation to treatment success (adjusted OR 0.98; 95 % CI 0.96–1.00). In addition, being female was associated with lower default rates in relation to treatment success (adjusted OR 0.57; 95 % CI 0.35–0.91).

**Table 2:** Multinomial logistic regression of factors associated with treatment outcomes in new TB patients

	Defaulted vs. Success^a^	Died vs. Success^a^	Defaulted vs. Success^a^	Died vs. Success^a^
	Unadjusted OR (95 % CI)	Unadjusted OR (95 % CI)	Adjusted OR (95 % CI)^b^	Adjusted OR (95 % CI)^b^
Age, years	0.98 (0.96–1.00)	1.03** (1.01–1.05)	0.98* (0.96–1.00)	1.04** (1.02–1.07)
Gender				
Male	Ref.	Ref.	Ref.	Ref.
Female	0.60* (0.38–0.94)	1.16 (0.62–2.19)	0.57* (0.35–0.91)	1.06 (0.55–2.06)
Type of TB				
EPTB	Ref.	Ref.	Ref.	Ref.
Smear-negative PTB	0.98 (0.54–1.77)	0.81 (0.36–1.79)	0.88 (0.48–1.62)	0.60 (0.26–1.39)
Smear-positive PTB	0.85 (0.49–1.49)	0.61 (0.29–1.32)	0.69 (0.38–1.26)	0.59 (0.26–1.34)
HIV status				
HIV-negative	Ref.	Ref.	Ref.	Ref.
HIV-positive on ART	0.70 (0.41–1.16)	0.72 (0.32–1.61)	0.71 (0.41–1.23)	0.80 (0.33–1.95)
HIV- positive not on ART	1.57 (0.88–2.81)	3.25** (1.53–6.91)	1.62 (0.88–2.97)	4.12** (1.76–9.66)

*Ref.* reference, *EPTB* extra-pulmonary tuberculosis, *OR* odds ratio, *95 % CI*, 95 % confidence intervals

* *p* < .05, ** *p* < .01, *** *p* < .001

^a^Success defined as cured and treatment completed

^b^Adjusted for age, gender, type of TB and HIV

Table 3 shows the findings of the multinomial regression analysis of the factors associated with treatment outcomes in HIV–positive TB patients. The odds ratio for death in relation to treatment success was found to be higher in patients HIV-positive not on ART (adjusted OR 7.38; 95 % CI 2.95–18.47) than in those HIV-positive on ART. Similarly, the odds ratio for default in relation to treatment success was found to be higher in patients HIV positive not on ART (adjusted OR 2.27; 95 % CI 1.15–4.47) than in those HIV positive on ART. In addition, increase age was associated with higher death rates in relation to treatment success (adjusted OR 1.05; 95 % CI 1.01–1.10).

**Table 3:** Multinomial logistic regression of factors associated with treatment outcomes in HIV-positive TB patients

	Defaulted vs. Success^a^	Died vs. Success^a^	Defaulted vs. Success^a^	Died vs. Success^a^
	Unadjusted OR (95 % CI)	Unadjusted OR (95 % CI)	Adjusted OR (95 % CI)	Adjusted OR (95 % CI)
Age, years	1.00 (0.97–1.03)	1.04* (1.01–1.09)	1.00 (0.96–1.03)	1.05* (1.01–1.10)
Gender				
Male	Ref.	Ref.	Ref.	Ref.
Female	0.74 (0.41–1.34)	1.08 (0.48–2.41)	0.75 (0.40–1.40)	1.65 (0.66–4.11)
Type of TB				
EPTB	Ref.	Ref.	Ref.	Ref.
Smear-negative PTB	1.21 (0.59–2.45)	1.13 (0.44–2.87)	1.05 (0.51–2.18)	0.74 (0.26–2.07)
Smear-positive PTB	0.97 (0.46–2.05)	0.92 (0.34–2.47)	0.85 (0.40–1.82)	0.68 (0.23–2.01)
CD4 count, cells/μL				
<50	Ref.	Ref.	Ref.	Ref.
50–199	0.73 (0.32–1.67)	0.43 (0.16–1.11)	0.79 (0.34–1.85)	0.42 (0.15–1.17)
200–349	0.88 (0.36–2.19)	0.31 (0.09–1.06)	0.84 (0.33–2.15)	0.20* (0.05–0.75)
≥350	1.20 (0.41–3.48)	0.39 (0.08–1.91)	0.84 (0.26–2.68)	0.14* (0.03–0.77)
ART status				
HIV-positive on ART	Ref.	Ref.	Ref.	Ref.
HIV- positive not on ART	2.26** (1.23–4.14)	4.53*** (2.03–10.10)	2.27* (1.15–4.47)	7.38*** (2.95–18.47)

*Ref.* reference, *EPTB* extra-pulmonary tuberculosis, *OR* odds ratio, *95 % CI* 95 % confidence intervals

* *p* < .05, ** *p* < .01, *** *p* < .001

^a^Success defined as cured and treatment completed

In patients with a CD4 count 200–349 cells/μL, compared to those with CD4 cell counts <50 cells/μL, the odds ratio for death in relation to treatment success was low (adjusted OR 0.20; 95 % CI 0.05 – 0.75). Similarly, the odds ratio for death in relation to treatment success was found to be lower in patients with CD4 count cell counts ≥350 cells cells/μL (adjusted OR 0.14; 95 % CI 0.03–0.77) compared to those with CD4 cell counts <50 cells/μL.

## Discussion

In this study, that assessed the impact of HIV, ART status and CD4 cell count on TB treatment outcomes among 797 new TB patients in a co-located ART and TB clinic, death and default rates were significantly higher among HIV-positive patients not on ART than HIV-negative patients. However, there were no differences in death and default rates between HIV-positive TB patients on ART and the HIV negative TB patients. ART uptake during the TB treatment episode was shown to significantly improve mortality and default rates for the HIV-positive TB patients.

Relatively poor uptake of ART has been reported in other studies on various forms of integrated ART and TB services and our finding that 28.2 % of 472 HIV-positive TB patients deemed eligible did not initiate ART during TB treatment in spite of co-location of TB and ART services lies within the range of 27.3 % and 62 % reported in sub-Saharan Africa programs providing fully integrated TB and ART services [12, 13, 19, 33] and is lower than the range of 66–86 % reported in geographically separate services [34–36]. Therefore, the barriers to successful uptake of ART seem common to all studies regardless of the model of care applied. These data clearly indicate that ART uptake should be improved within this setting of co-located TB and ART services or that there is a requirement for a different model of integration of services that would provide for a closer monitoring of ART uptake in patients with HIV-associated TB. Owing to the retrospective design of this study, the barriers to ART uptake were unknown.

Our study finding that ART uptake during TB treatment was significantly associated with improved mortality rates, is similar to a study among patients accessing an integrated TB and ART service in Malawi [19] and is in keeping with evidence that ART during TB treatment improves survival from a randomized controlled trial conducted in South Africa [37]. The study findings call for the strengthening of HIV/TB collaborative activities to ensure efficient treatment integration, which could lead to improved TB treatment outcomes HIV-positive TB patients. However, this community health centre has two health authorities rendering either TB or ART services that are not harmonized owing to organizational differences. Therefore, there is a need to unify these separate health authorities at all levels of the health system (which range from clinical care to community support and administrative structures) in order for the community health centre to ensure the successful delivery of integrated TB and ART care for HIV-positive TB patients.

Similar to other studies from sub-Saharan Africa and elsewhere [5–8, 37–45], we found that higher CD4 cell counts (≥200 cells/μL) were associated with reduced mortality, an indication that patients with CD4 counts <200 cells/μL) should be fast-tracked for ART. This, however, may only be achieved if ART is prescribed and administered by the TB services.

The strength of this study is that it provides an insight into the HIV, ART status and CD4 cell count on TB treatment outcomes among new TB patients attending a TB service co-located within the same facility as an ART service, although the two services remain separately staffed. The study findings, therefore, are useful to inform policy and programs that aim to improve TB treatment outcomes among new patients with HIV-associated TB in this TB service and other comparable settings.

The limitations of the data used in present study must be acknowledged. We used only routinely collected program data which did not have details of HIV-RNA viral loads of patients and antiretroviral treatment histories of patients already on ART at the time of TB diagnosis (such as antiretroviral resistance, virological treatment failure and clinical immunological failure). The study, therefore, could not explore the effect of these factors on TB treatment outcomes. In addition, non-randomized studies are subject to possible residual confounding bias by unmeasured confounders. Due to small numbers, we could not explore the effect of duration on ART or time delay of ART initiation on treatment outcomes. A further limitation of our study was that the routinely collected program data available did not have details regarding TB culture results or the distribution of drug resistant versus drug sensitive forms of TB in the study population. Therefore, the study could not determine the influence of these factors on TB treatment outcomes.

## Conclusions

In conclusion, this study showed that death rates were similar for HIV-positive TB patients on ART and HIV-negative TB patients but were considerably higher among HIV-positive patients not on ART. Moreover, despite the co-location of TB and ART services in the same building 28.2 % of 472 HIV-positive TB patients who were eligible to start ART did not initiate ART during the TB treatment episode. These patients had significantly higher death rates and default rates. These findings stress the need for an intervention aimed at improving ART uptake targeting both patients and health care providers.

## Abbreviations

TB: Tuberculosis
HIV: Human immunodeficiency virus
ART: Antiretroviral therapy
